# A Native Human Monoclonal Antibody Targeting HCMV gB (AD-2 Site I)

**DOI:** 10.3390/ijms19123982

**Published:** 2018-12-11

**Authors:** Michael M. McVoy, Edgar Tenorio, Lawrence M. Kauvar

**Affiliations:** 1 Department of Pediatrics, Virginia Commonwealth University School of Medicine, P.O. Box 980163 MCV Station, Richmond, VA 23298-0163, USA; michael.mcvoy@vcuhealth.org; 2 Trellis Bioscience, LLC, 702 Marshall Street, Suite 614, Redwood City, CA 94063, USA; etenorio@trellisbio.com

**Keywords:** cytomegalovirus, cell tropism, conserved site, monoclonal antibody, neutralization, congenital transmission, immune suppression

## Abstract

Hyperimmune globulin (HIG) has shown efficacy against human cytomegalovirus (HCMV) for both transplant and congenital transmission indications. Replicating that activity with a monoclonal antibody (mAb) offers the potential for improved consistency in manufacturing, lower infusion volume, and improved pharmacokinetics, as well as reduced risk of off-target reactivity leading to toxicity. HCMV pathology is linked to its broad cell tropism. The glycoprotein B (gB) envelope protein is important for infections in all cell types. Within gB, the antigenic determinant (AD)-2 Site I is qualitatively more highly-conserved than any other region of the virus. TRL345, a high affinity (K*d* = 50 pM) native human mAb to this site, has shown efficacy in neutralizing the infection of fibroblasts, endothelial and epithelial cells, as well as specialized placental cells including trophoblast progenitor cells. It has also been shown to block the infection of placental fragments grown ex vivo, and to reduce syncytial spread in fibroblasts in vitro. Manufacturing and toxicology preparation for filing an IND (investigational new drug) application with the US Food and Drug Administration (FDA) are expected to be completed in mid-2019.

## 1. Introduction

### 1.1. Epidemiology and Clinical Need

Human cytomegalovirus (HCMV) infects 60–70% of adults in industrialized countries and nearly 100% of adults in emerging countries [1]. In the US, about 60% of people are infected by age 6 and 85% by age 80 [2]. Most infections are benign or resolve naturally. The most common medically-significant infection is in transplant patients [3], strongly interfering with transplant success. HCMV disease is characterized by fever and malaise as well as leukopenia, thrombocytopenia, and elevated liver enzymes; upper digestive tract symptoms, mainly pain, are also common. As a leading cause of post-transplant hospitalization, HCMV contributes substantially to the high cost of this effective therapeutic option. In the US, there are ~25,000 solid organ transplants annually. An additional ~20,000 patients receive a hematopoietic stem cell transplant (HSCT) annually. With improvements in diagnosis and antiviral intervention, the incidence of early onset HCMV disease has been reduced to <5%, although the late onset disease is still a significant problem [4,5]. With improving prognoses [6], it is likely that more transplants will be conducted, particularly for hematopoietic stem cells which are available from bone marrow in a renewable fashion. 

In addition, HCMV is the leading cause of congenital viral disease [5]. Among live births, more babies are affected by HCMV infection in utero than by Down syndrome, by fetal alcohol syndrome, or by neural tube defects [7]. Primary HCMV infection during early pregnancy poses a 30–40% risk of intrauterine transmission. Of these congenitally-infected infants, 10–15% are symptomatic, presenting with intrauterine growth restriction and permanent birth defects, including neurological deficiencies, retinopathy and sensori-neuronal deafness; of the infected but asymptomatic infants, 15–20% will later develop permanent sequelae [4,8,9,10]. Infection at an early gestational age increases the severity of these problems [10]. Even secondary reactivations or re-infections of seropositive (previously exposed) women can lead to birth defects if the virus is passed on to the fetus [11]. HCMV infection impacts placental growth leading to intrauterine growth restriction. The associated medical costs at delivery are significant, with further costs associated with cardiovascular disease that develops later in life [12]. Moreover, congenital HCMV is linked to ~15% of stillbirths (death in utero after 20 weeks of gestation) [13,14]; infection of the cervix also contributes to preterm labor [15]. Infected placentas show pathological changes that undermine transport functions [16]. Of particular concern is infection of trophoblast progenitor cells (TBPCs) in the chorionic membrane. These stem cells differentiate into the mature trophoblast subtypes (transport syncytiotrophoblasts and invasive cytotrophoblasts), and their continued replication, self-renewal, and differentiation are critical for the formation of new chorionic villi, the functional units of the placenta. HCMV infection blocks TBPC differentiation and fusion into syncytiotrophoblasts [17]. Overall, ~120,000 pregnancies annually in the US are at high risk from HCMV infection. Screening of mothers for signs of HCMV infection is not routine in the US, but has been widely adopted in Europe [18]. 

### 1.2. Genome and Proteome

HCMV, also known as human herpesvirus 5 (HHV-5), has a double-stranded DNA genome of 235 kilobase pairs, making it one of the largest human viruses. Over 170 open reading frames have been identified encoding proteins larger than 100 residues, including 20 proteins associated with the virion envelope [19]. Only some of these proteins have been well characterized in terms of function. In addition, next generation sequencing has revealed high levels of transcription from non-coding and antisense regions. Further, within the coding regions, splicing patterns were found to be more numerous and complex than previously appreciated. Finally, numerous smaller peptides have also been identified, most of which are of unknown function [20]. 

Broad cell tropism is a distinguishing feature of HCMV compared to other virus families [21]. Inter-host transmission is enhanced by infection of epithelial cells. Intra-host spread is enhanced by infection of endothelial cells and hematopoietic cells. Infection of ubiquitous fibroblasts provides a platform for efficient proliferation of the virus. 

The glycoprotein B (gB) envelope protein provides the cell fusion activity required for infection of all cell types; other envelope proteins are implicated in infectivity of specific cell types. A dimer of glycoproteins H and L (gH/gL), in complex with other proteins, plays a modulatory role on cell tropism. A “trimer” comprised of gH/gL complexed with the heavily glycosylated glycoprotein O (gO) interacts with gB to promote membrane fusion and viral entry into all cell types [22], while an alternative “pentameric complex” of gH/gL complexed with subunit proteins UL128, UL130, and UL131A specifically facilitates infection of epithelial and endothelial cells [23,24]. Serial passage of HCMV in fibroblasts in vitro leads to mutations that disrupt pentameric complex expression in favor of the trimer [25]. 

A recently described viral protein localized to the endoplasmic reticulum, UL148, appears to promote incorporation of the trimer into virions. Although substantially elevated pentamer is normally needed for entry into epithelial cells, a UL148-null variant of viral strain TB40/E showed enhanced epithelial cell tropism; that effect appears to reflect defective trimer formation rather than an increase in pentamer levels. This suggests that the absolute level of the pentameric complex does not regulate tropism for epithelial cells as much as the ratio of trimer to pentamer [26,27].

Viewed from a perspective of drug targeting, gB is of interest since it plays a role in the infection of all cell types. By comparison, targeting the pentameric complex may have reduced impact on suppressing viral load. A further favorable feature of gB is that it is qualitatively more conserved than the rest of the HCMV genome. Using next generation sequencing technology to determine single nucleotide polymorphisms (SNP) for the ensemble of HCMV viral genomes from single individuals, the frequency of variant amino acids at each position across >1000 viral genomes from a single individual ranges from 0.1–0.3% [28]. The immunodominant gB antigenic determinants, AD-4 and AD-1, are significantly more variable than the much less immunogenic AD-2, for which the frequency of natural substitutions is approximately 10-fold lower (across 17 genomes from different strains), with 8 out of 22 observed substitutions being highly conservative (T > S, Y > F, I > F) [29]. The AD-2 site is the target for the mAb TRL345 discussed in detail below. 

## 2. Therapeutic Approaches

### 2.1. Small Molecules

Ganciclovir, a nucleoside analog (or its prodrug valganciclovir), has become the standard of care in management of HCMV in the transplant setting [15]. This nucleoside analog inhibitor of the viral polymerase has strong neutropenic activity, which is of particular concern early after hematopoietic stem cell transplant, when the risk of HCMV infection is most serious. As a potential teratogen, it is also not appropriate for use in the congenital transmission indication. Mutations leading to resistance to ganciclovir have been well characterized, and are a known risk factor for poor outcomes in transplant patients [30]

The majority of post-transplant HCMV disease now occurs after discontinuation of ganciclovir. In a recent randomized double blind trial of valganciclovir (*N* = 95) vs. placebo (*N* = 89), prophylaxis for 6 months was no better than preemptive therapy (initiation of antiviral treatment when viral burden in blood exceeded 1000 genome copies/mL measured by a qPCR assay). For both cohorts, ~20% of patients met the composite primary endpoint of death or HCMV disease by day 270 post-transplant [31]. In addition to fever, malaise, leukopenia, thrombocytopenia and elevated liver enzymes, HCMV disease is commonly characterized by upper digestive tract pain. Mortality is generally associated with multi-organ dysfunction including pneumonia. 

Although ganciclovir has been a successful drug, there remains a significant unmet medical need for new therapeutics to treat HCMV disease [32]. Letermovir was approved in November 2017 for prevention of HCMV infection and disease in adult HSCT patients [33]. This non-nucleoside small molecule drug was developed in part to address the polymerase drug resistance issue by inhibiting a different viral target contributing to HCMV replication [34], namely, the terminase DNA packaging and cleavage complex, and in part to improve on the efficacy and toxicity profiles of the polymerase inhibitors [35]. Letermovir efficacy was established in a Phase 3 trial of seropositive recipients of HSCT (*N* = 495 without detectable HCMV DNA at randomization) [36]. The primary endpoint was the proportion of patients who had clinically-significant HCMV infection through week 24 after transplantation: 37.5% in the treated group vs. 60.6% in the placebo group (*p* < 0.001). Adverse events with letermovir were mainly of low grade. A particularly favorable feature compared to ganciclovir is the lack of neutropenia. However, a significant concern regarding this drug is that escape mutations are readily generated in vitro [37,38], with several examples reported of resistance emerging during treatment [39,40]. For example, a recent case study reported on a lung transplant recipient with a ganciclovir-resistant HCMV infection; treatment with letermovir showed efficacy in the initial 5 weeks of treatment, but a resistant mutant arose over the subsequent 10 weeks of treatment [41].

Other small molecule drugs have shown disappointing efficacy. Maribavir, which inhibits capsid nuclear egress, initially failed to prevent HCMV disease in HSCT patients [42], but is currently undergoing Phase 3 evaluations at higher doses [43,44]. Brincidofovir, a prodrug form of cidofovir (a nucleotide analog polymerase inhibitor), was expected to be more potent and less toxic than cidofovir, but it failed to meet its efficacy endpoints in Phase 3 trials [45].

An unusual new approach to HCMV therapy is based on the observation that the gH/gL/gO trimer uses the PDGF (platelet-derived growth factor) receptor for cell entry [46]. In a single arm Phase 2 trial, a receptor kinase inhibitor (nilotinib, an approved drug for treatment of chronic myeloid leukemia), suppressed HCMV infection in 80.6% of HSCT patients (*N* = 37) [47].

### 2.2. Vaccines

To date, vaccination to prevent HCMV infection has not been proven to provide adequate protection against maternal infection, re-infection, or fetal transmission [48,49]. Vaccination is difficult to implement in immunosuppressed patients, notably including cancer patients, who are the major recipients of bone marrow transplants. 

Recombinant gB formulated with MF59 adjuvant showed an approximately 50% reduction in primary maternal infections in vaccinated women, supporting a focus on gB as a target [50]. Unfortunately, the conserved epitope on gB (AD-2, site I) is poorly immunogenic [51,52], which may have contributed to the failure to protect a larger percentage of the population. 

The same vaccine has also shown activity in solid organ transplant [53]. In this study, 67 patients received vaccine and 73 a placebo, with gB titer being substantially increased in both seronegative and seropositive subjects (*p* < 0.0001). In those who developed viremia after transplantation, anti-gB antibody titers correlated inversely with duration of viremia (*p* = 0.002). In seronegative patients with seropositive donors, the duration of viremia (*p* = 0.05) and number of days of ganciclovir treatment (*p* = 0.03) were reduced in vaccine recipients.

T cell responses appear to be more important for suppressing latent infection than for blocking initial infection. Augmenting the T cell response by vaccination must contend with the large fraction of most individuals’ T cell repertoire that is already directed against HCMV-encoded epitopes, in some cases in excess of 40% of the entire CD4+ cell response [54]. A DNA plasmid-based vaccine (ASP0113, Astellas /Vical) included pp65 as a T cell determinant and gB as a B cell determinant, but titer to gB was weak [55] and the company announced in January 2018 that it had failed in a Phase 3 trial in the HSTC indication. 

An alternative to traditional vaccination for stimulating T cells is ex vivo stimulation with HCMV antigens, which has shown promise in the HSCT indication [56], but this technology is cumbersome to implement at commercial scale and has only been tested in sibling transplants for treatment beginning at 27 days post-transplant to avoid problems in engraftment of the transplant. Half of all HCMV outbreaks in this population occur within the first 29 days, a significant impediment to widespread use of the therapy.

### 2.3. Hyper Immune Globulin (HIG)

An alternative to active vaccines for stimulating a polyclonal B cell response is to administer HIG, which has been used safely for decades in pregnancy to treat blood group incompatibilities, rubella, hepatitis, varicella, and measles [57]. Passive HIG immunization had shown promising results in human clinical trials from 2005 to 2012 [58,59,60,61,62], although the degree of efficacy became controversial when a 2014 study [63] failed to meet the primary endpoint of preventing fetal infection. However, limitations of this study included recruitment as late as 26 weeks and a median interval between diagnosis of infection and first HIG administration of five weeks. The study was thus under-powered and a meta-analysis of the data revealed that efficacy was in fact likely at *p* < 0.05 for first trimester mothers who received prompt treatment [64]. 

Subsequent study of the pharmacokinetics of HIG has revealed that the high dose of IgG given to pregnant women results in faster clearance (11 days) than what is familiar for numerous monoclonal antibodies (22 days) [65]. Based on this important observation, a small trial was conducted [66]. Subjects were 40 pregnant women with a primary HCMV infection diagnosed at a median gestational age of 9.6 weeks. On average, bi-weekly HIG administration started at 11.1 weeks and continued until 16.6 weeks’ gestation (2–6 doses). In all cases, amniocentesis was performed at least 6 weeks after the first presentation, in general at about 20 weeks. The neonates were also tested for viral DNA after birth. Maternal-fetal transmission before amniocentesis occurred in only 1 of the 40 cases (2.5%). At delivery, 2 additional subjects were found to have had later gestation transmission. Considering all three cases of maternal-fetal transmission, the transmission rate was 7.5%. Importantly, none of the infected neonates were symptomatic at birth. The results were compared with two untreated historical cohorts of first trimester primary infections that had an amniocentesis at about 20 weeks. In the combined historical control cohort of 108 pregnancies, there were 38 transmissions (35.2%), which was significantly higher than in the HIG administration group (*p* < 0.0001). Moreover, in the untreated historical cohort, the incidence of sequelae in the infected infants was 14%, which stands in sharp contrast to the absence of symptomatic infants in the bi-weekly HIG-treated group.

HIG has also been widely used to prevent HCMV infection in solid organ transplant, both alone and in combination with antivirals [67]. A meta-analysis of 11 randomized trials (*N* = 698; median follow-up: 12 months) concluded that prophylactic administration of HIG was associated with improved total survival, reduced HCMV disease and HCMV-associated mortality [68]. In a recent study of pediatric HSCT, HCMV infection at 1 year was 13.4% for HIG-treated vs 44.4% with no HIG (*p* = 0.001) [69]. 

Vaccines and polyclonal sera provide the foundation for discovery of single mAbs or combinations of mAbs as clinical agents, as summarized in Table 1 with details provided below. 

### 2.4. Monoclonal Antibodies (mAbs)

Several reports have described mAbs that neutralize HCMV [49]. An extensive group of published mAbs is directed against the adjunct proteins that modulate gB activity (gH, gL, UL128, UL130, UL131A) [24,70,71]. Anti-pentameric complex antibodies are abundant in commercial HIG [72] and are made early in the course of natural infection [73], although immunodominance is not necessarily correlated with functional importance. In fact, given the long association of HCMV with humans (estimated to be over 100,000 years), the immunodominant epitopes may have evolved in order to misdirect the immune system away from curative responses. Since HCMV infection tends to be lifelong, with latent infections now understood to involve slow replication [74] and not a completely dormant state, it is clear that the natural immune response is not curative. A drawback to targeting the pentameric complex is that antibodies against it fail to neutralize infection of fibroblasts [75], whose ubiquitous distribution in the body may make them an important cell type for disease progression. In particular, virus passaged in fibroblasts can infect both fibroblasts and endothelial cells whereas virus released from endothelial cells readily infected fibroblasts but was barely able to infect endothelial cells [76].

The major clinical mAb effort thus far has been RG7667 (Genentech) which is a mixture of two mAbs; the first is a human-derived mAb specifically targeting gH; this mAb is a derivative of MSL-109, which had earlier failed in clinical trials [45] possibly due to an unusual propensity for rapid development of resistance through selective uptake by infected cells and incorporation into assembling virions [77]. The second component is a humanized murine mAb that targets the pentameric complex. Both mAbs are reasonably high affinity, and in a Phase 2 trial in renal transplant patients, HCMV disease was less common in the RG7667 group than the placebo group (3.4% versus 15.8%; *p* = 0.03), and time to viremia was delayed [78]. A comparison PK of the individual mAbs in each treated individual indicated that high exposure to both mAbs had better activity than high exposure to only one [79]. Although these results are promising, this product is no longer listed in Genentech’s pipeline. 

An extensively studied group of mAbs is directed towards gB [80], which has been a key component of all subunit vaccines to date. CSJ148 (Novartis) is a mixture of 2 mAbs; the first targets gB (AD-4), an immunodominant region; the second targets the pentameric complex [81]. In a Phase 1 trial, CSJ148 was safe and well tolerated, with pharmacokinetics as expected for human immunoglobulin [82]. Following a Phase 2 trial in HSCT, a Phase 2 study for preventing congenital transmission was listed early in 2018, although this trial was withdrawn in August 2018 prior to enrollment following a change in prioritization of infectious disease products within the company. TRL345, described below, is ~6.5-fold more potent than the anti-gB component of CSJ148, averaged across assays using multiple cell types [80,83].

Both AD-4 and the similarly immunodominant AD-1 are significantly more variable than the much less immunogenic AD-2 Site 1. While > 90% of mAbs to the immunodomimant regions are not neutralizing [84], higher titer to the AD-2 epitope is associated with reduced risk of congenital transmission [85], with an odds ratio of 0.72 (*p* = 0.05) for every 0.5 log_10_ increase in titer. In this study, which included a panel of HCMV glycoprotein complexes, anti-gB (AD-2) was the only specificity that had a statistically-significant impact on transmission risk. This study examined untreated, HIV-infected women and their infants in the US from the Women and Infants Transmission Study (WITS). A multivariable logistic regression model was performed with correction for factors previously associated with the risk of congenital infection including: race, maternal age and parity. Results were adjusted for maternal peripheral CD4+ T cell counts and HIV virus load. 

TCN-202 (Theraclone), a human mAb targeting the conserved gB (AD-2 Site I) epitope was discontinued following Phase 1 after a proposed corporate merger effort failed. This mAb has similar specificity as TRL345 (described below), but is ~10-fold less potent.

Affinity is an important consideration in developing a protective mAb against HCMV, particularly for the congenital indication. Weak affinity is associated with enhanced fetal transmission [86], possibly because only high affinity (slow off-rate) mAbs remain bound to the virus following transcytosis across the placenta via the neonatal Fc receptor [87]. Variability in affinity for different antigens may also result in variable efficacy of different batches of HIG, which are not optimized for high titer, or even uniform titer, to any epitope. For mAbs targeting gB, another reason to seek high affinity is that competitive binding between neutralizing and non-neutralizing antibodies is a normal part of the natural immune response to gB [88]. High affinity of the neutralizing mAb reduces interference by mAbs binding with weaker affinity [51]. The potential impact is substantial since non-neutralizing antibodies to Site II on gB (AD-2), adjacent to Site I, were found in ~25% of anti-HCMV human serum samples [89]. 

## 3. TRL345: A Clinical Candidate

### 3.1. Discovery

Trellis’s proprietary CellSpot™ platform [90,91] uses digital microscopy to examine the secreted IgG footprint from each of millions of memory B cells. In an initial survey prior to the cloning effort, we observed wide variation in anti-gB titer. From 5 million B cells surveyed, drawn from 48 donors, with an emphasis on the 4 donors who had the highest titer to gB (AD-2), 30 mAbs were cloned by single-cell cDNA PCR and expressed in HEK293 cells. Based on superior potency for neutralization of HCMV in vitro [83], TRL345 was chosen as the lead candidate [92].

Immunization with peptides from the AD-2 site induces reasonable titer (in mice or rabbits) but the resulting antibodies have poor neutralizing activity compared to using the full protein as immunogen [93], suggesting conformational character to the epitope. All of the 30 mAbs cloned from the highest titer human donors use a very restricted set of germ line variable domain genes: IGHV3-30 and IGKV3-11 [83]. This overwhelming preponderance of a single VH and VL family, out of more than 10^6^ possibilities [94], may reflect structural constraints described in a study showing that these germline V-genes encode key side chain contacts with the viral antigen [95]. Similar preponderance of a restricted set of germline sequences has previously been described for both dengue and influenza viruses [96,97], although the HCMV mAb repertoire is even more restricted than the influenza repertoire against the highly conserved stalk region [83]. We speculate that random initiation of mAb maturation from outside this restricted set of genes may have contributed to the efficacy of the gB/MF59 vaccine only reaching the 50% level [50]. Table 2 summarizes these properties of TRL345 along with further biochemical and bioassay data described in more detail below.

### 3.2. Biochemical Properties

TRL345 binding was measured by ELISA against a set of overlapping sequence peptides derived from the AD-2 sequence, identifying the epitope as residues 69 to 77 (ETIYNTTLK) corresponding to the highly-conserved AD-2 Site I [92]. An epidemiology study in Japan identified titer to this epitope as a key independent predictor of clinical status of renal transplant patients. Those with higher titer had lower incidence of HCMV infections requiring adjunct antiviral therapy [98]. 

Antibodies with the specificity of TRL345 are found in sera from naturally infected subjects, representing ~1% of the anti-HCMV repertoire [99]. Thus, there has historically been selection pressure on the virus acting on the normally expected frequency of mutations at this site. The actual frequency of such mutations is ~10-fold lower than for other envelope glycoprotein sites [28,29], suggesting that escape from TRL345 will be rare. 

Kinetics of binding to the immobilized AD-2 peptide yielded a K*d* of ~1 pM, with a somewhat higher K*d* (~50 pM) for the gB protein, possibly reflecting sequence polymorphisms elsewhere in the gB protein that alter its conformation, and thereby influence the ability of TRL345 to bind its epitope, or greater ease of induced-fit binding for the more flexible peptide. 

### 3.3. Potent Neutralization across Broad Spectrum of Cell Types 

To determine if TRL345 was effective against HCMV clinical strains, neutralizing assays were conducted using 15 primary clinical isolates of diverse genotypes [100]. TRL345 prevented infection of MRC-5 fibroblasts by all 15 isolates [92]. 

Using the HCMV strain VR1814, which is genetically more authentic as compared to attenuated strains, TRL345 was effective in preventing infection of human endothelial cells, smooth muscle cells, placental fibroblasts, and trophoblast progenitor cells. For comparison, a high affinity anti-pentameric complex antibody was cloned from published sequence data (mAb 1F11 binding to UL128-131A) [75,101]. This mAb had more potent neutralizing activity on endothelial cells than TRL345, but it provided no protection against infection of smooth muscle cells, placental fibroblasts, or trophoblast progenitor cells [92]. TRL345 was 50-fold more potent than HIG using VR1814 to infect MRC-5 human lung fibroblasts. A 10-fold potency advantage over HIG was observed using GFP-tagged epithelial-tropic variants of the laboratory strains Towne (TS15-rN) and AD169 (BADr) to infect MRC-5 fibroblasts and ARPE-19 epithelial cells, respectively. 

Young children shedding virus (saliva and urine) are the main vector of maternal infections. To be successful, maternal vaccination needs to target the most relevant viral proteins. Primary urine derived HCMV (uCMV) infects fibroblasts much more efficiently than epithelial cells, although even a single passage in fibroblasts is sufficient to improve efficiency of epithelial cell entry [102]. These results may indicate that fibroblasts act as a launching platform for virus able to infect other cell types throughout the body. Primary uCMVs are highly resistant to antibody neutralization. Seven potent neutralizing mAbs against diverse antigens were assessed for uCMV neutralization. Only TRL345 showed partial activity, albeit at a high concentration (50 µg/mL). Elucidating the dissemination route, and whether fibroblast infection is important in that process, is an area of active research [103]. 

Preventing entry of virus into cells is an important component of natural immunity. However, cell-to-cell spread may also be important parameter. Formation of multi-nucleated syncytia is a characteristic phenotype of HCMV in vivo. In vitro HCMV cell-to-cell spread is largely resistant to antibodies in fibroblast cultures but sensitive in endothelial and epithelial cell cultures. A recent study of viral entry and syncytial spread in vitro on a range of cell types profiled a comprehensive panel of 28 antibodies, both polyclonal and monoclonal, targeting epitopes in HCMV virion glycoprotein complexes including gB, gH/gL, and the pentameric complex [104]. While all antibodies that neutralized epithelial cell entry also inhibited spread in epithelial cells, with well-correlated potencies of these two activities, none fully inhibited syncytial spread in fibroblasts. The most effective antibody was TRL345, which prevented the formation of large syncytium-like structures albeit at a concentration ~100-fold higher than needed for neutralizing fibroblast entry. An additional observation from this study was that antibodies targeting epitopes in gH or gH/gL exhibited strain- and cell-type-dependent neutralizing activities that implicate polymorphisms both within and outside gH/gL in resistance to neutralization.

### 3.4. Ex vivo Model for Congenital Indication

HCMV is entirely species-specific, i.e., it is restricted to infecting human cells. The resulting lack of a validated animal model has put a premium on studies using human placental tissue as a key model system. HCMV replicates in the TBPCs and impairs the capacity to self-renew and differentiate [17]. TBPCs differentiate into several cell types critical for placental function. HIG is about equally effective at blocking infection by VR1814 of TBPC and ARPE-19 (epithelial cells). TRL345 was equally active against entry into both cell types, with ~10-fold superior potency compared to HIG. By contrast, two mAbs against the pentameric complex showed negligible activity on TBPCs despite being quite active on ARPE-19 cells [16]. 

To further characterize TRL345 neutralizing activity, it was tested in an established ex vivo model in which explants of first trimester human placenta are grown on Matrigel [105]. Within 24 h, the villus cytotrophoblasts proliferate and differentiate, forming cell columns that develop further into anchoring villi that attach the explant to the Matrigel substrate. VR1814 incubated with an isotype-matched negative control mAb readily infected cell columns. Incubation with HIG showed a trend towards efficacy, but did not reach statistical significance for the median percentage of HCMV-positive cells. TRL345 was highly effective at reducing infection (*p* < 0.05 vs HIG, and *p* < 0.01 vs isotype control). The bioequivalent dose (defined by neutralization EC50) of the monoclonal TRL345 was 50-fold lower than for the polyclonal HIG, consistent with only 1–2% of HIG being against gB.

### 3.5. IND-Enabling Studies

TRL345 is a native human mAb that resulted from extensive somatic mutation. A total of fifteen residues in the CDRs (complementarity determining regions) were mutated in TRL345 in comparison to germ line V segments, including eleven in the heavy chain and four in the light chain [92]. None of these mutations represent new stability risks such as motifs for oxidation or deamidation, isomerization, glycosylation, or acidic cleavage. TRL345’s melting temperature was 70°C. Expression at 1.8 g/liter in stably-transformed CHO cells has been established using the GPEx™ expression system (Catalent Pharma Solutions; Madison, WI, USA). A non-GMP toxicology lot (100 L scale) was successfully completed, and a 250 L scale GMP lot is underway. TRL345 was also evaluated for tissue reactivity to a panel of cryosections of 38 adult and 21 fetal (0 to 6 months) human tissue samples (Charles River Laboratories; Reno, NV, USA). TRL345 binding was observed on epithelial cells in several adult (colon, skin, small intestine, and pancreas) and fetal (small intestine and pancreas) human tissues but only in samples from one or two of the three donors. An extensive survey of cell surface-expressed human proteins failed to identify a cross-reactive antigen. Due to the widespread prevalence of HCMV infection in humans, the observed binding in some instances may have been due to HCMV latent infections which are now understood to involve slow replication [74].

A 28-day toxicology study in Sprague Dawley rats has been completed. There were no TRL345-related, toxicologically significant effects on any of the parameters evaluated. Therefore, the no-observed-adverse-effect-level (NOAEL) was considered to be 150 mg/kg/dose.

The effective dose of TRL345 in vitro ranges from 0.5 µg/mL for inhibiting VR1814 cell entry in a variety of cell types to 50 µg/mL for inhibiting fibroblast infection by urine-derived virus and syncytial spread in fibroblasts. Based on the pharmacokinetics measured in rats, and comparison to other human mAbs characterized in clinical trials, a TRL345 dose in humans of 15 mg/kg is expected to have a half-life of 21 days, with serum level exceeding 50 µg/mL for 7–10 days.

## 4. Discussion

An ideal treatment for HCMV infection should protect against infection by the full range of circulating strains, while further addressing the broad cell tropism of this virus. Targeting the viral replication system is generally cell-type independent, and is typically achieved by small molecule drugs, most of which have been nucleoside analogs which are problematic for the congenital indication due to potential teratogenicity. Further, drug resistance has been a recurring issue for this class of drugs. 

As an alternative, or complementary, approach HIG has shown persuasive activity for both transplant and congenital indications. To achieve comparable efficacy by more readily manufactured agents, several viral envelope antigens have been proposed as targets for vaccines or therapeutic mAbs. A key advantage of a mAb over HIG is higher potency allowing lower infusion volumes. In addition, HIG includes a vast majority of antibodies with unknown antigen specificity; for that reason, a mAb offers less risk of off-target reactivity contributing to toxicity. The advantages of a mAb over vaccines include more reliable and more rapid achievement of a neutralizing concentration, particularly in immune compromised subjects; that is, the response of each host’s immune system is idiosyncratic and might not generate a therapeutically adequate immune response. 

Antibodies to the pentameric complex, including gH/gL and its further complexes with gO and UL128-131A, dominate the neutralizing immune response. This natural response is largely effective at keeping HCMV levels low in healthy individuals, and Genentech has focused its HCMV effort on a mixture of 2 mAbs targeting pentameric complex proteins (RG7667). Following the Phase 2 randomized trial results reported in 2017, this product is no longer listed in Genentech’s pipeline. 

Antibodies to the gB fusion protein are also part of the natural immune response. This target is of specific interest because it is essential for entry in all cell types, not just those for which pentameric complex interactions regulate cell tropism. An adjuvanted gB vaccine has shown efficacy in a substantial fraction of subjects, both in transplant and congenital indications. Within the gB protein, the AD-2 Site I is qualitatively more conserved than the rest of the gB protein (which is highly conserved overall), indicative of a critical function. However, this site is also poorly immunogenic, which may account for the gB/MF59 vaccine having achieved only partial efficacy. The first mAb to be tested clinically against this epitope, TCN-202, was safe and well tolerated in a Phase 1 trial, but dropped from development following a change in corporate priorities. The safety profile of this mAb is consistent with the favorable safety record of HIG which includes mAbs of similar specificity. 

TRL345 is a native human mAb that binds to gB (AD-2 Site I) with an approximately 10-fold tighter binding affinity than TCN-202. TRL345 has shown potent antiviral activity for a representative sampling of clinical isolates. For several clinical-like viral strains, it has shown protective activity for a wide range of cell types with potency 10-50 fold better than HIG. IND-enabling development is nearly concluded, with a production cell line in place and toxicology studies completed with no significant issues. 

The traditional clinical development path for HCMV drugs has focused on transplant indications, initially with renal transplant as the leading indication, but more recently with a focus on HSCT. The rationale has been that the risk of adverse events is more justifiable for these patients than for pregnant women. HIG has shown efficacy in both transplant and congenital indications, and mAbs drawn from the natural immune response pose lower risk for the congenital indication than other classes of drugs, enabling a shift in priorities. For example, after a small Phase 2 clinical study of CSJ148 in HSCT, Novartis announced plans to proceed to the congenital indication in the fall of 2018; this product is a mixture of 2 mAbs with the first targeting gB (AD-4) and the second targeting the pentameric complex. Due to a change in corporate strategic priorities disfavoring infectious dieseases, however, this trial was withdrawn from the clinicaltrials.gov website before being initiated. TRL345 has ~6.5-fold better affinity to the target protein than the anti-gB component of CSJ148; in addition the gB (AD-4) epitope is substantially less conserved than the gB (AD-2 Site I) epitope.

In light of the success of letermovir in the transplant indication, accruing patients for a HSCT trial has become more difficult. If drug resistance to letermovir proves to be clinically significant, as suggested by preclinical data and three early case studies, there will be increased motivation for evaluation of TRL345 in HSCT. In parallel, the improvement in outcome for the congenital indication when HIG was given early in pregnancy at a dose appropriate to the measured pharmacokinetics has stimulated interest in a mAb effort to reduce the sequelae of congenital transmission. Assessing the efficacy of TRL345 as a single agent is important before attempting combinations of mAbs to replicate the efficacy of HIG, a point emphasized by Genentech in their analysis of the complicated PK of their 2 mAb mixture [79]. The safety and efficacy of the gB/MF59 vaccine provides support for such a single agent study.

## Figures and Tables

**Table 1:** Status of HCMV vaccines and antibody-based therapeutics with clinical data.

Vaccine/mAb	Key Features	Status
Towne (Wistar)	Attenuated live virus, lacking pentameric complex	Effective in transplant but not in congenital
gB/MF59 (Sanofi)	Recombinant subunit vaccine with adjuvant	Effective in ~50% of subjects
Cytotect™ (Europe) Cytogam™ (US)	Hyper Immune Globulin (HIG) has shown efficacy in both transplant and congenital indications	Marketed products
ASP0113 (Vical, Astellas)	2 plasmid DNA vaccine (pp65, gB) induced only weak responses to gB	Development suspended after Phase 3
MSL-109 (Sandoz)	Binds gH, but efficacy is reduced by uptake of Ab-Ag complex into cells	Development suspended after Phase 3
RG7667 (Genentech)	2 mAbs, one targeting gH (similar to MSL-109), and one targeting the pentameric complex	Development suspended after Phase 2
CSJ148 (Novartis)	2 mAbs, one targeting gB (AD-4), and one targeting the pentameric complex	Development suspended after Phase 2
TCN-202 (Theraclone)	Human mAb against gB (AD-2, Site I)	Development suspended after Phase 2

**Table 2:** Properties of TRL345.

TRL345 Properties	Comments
Source	Native human mAb from anonymized blood
Isotype	IgG1, κ
Epitope	HCMV envelope glycoprotein gB (AD-2 Site I)
Affinity	K*d* = 50 pM for gB protein; K*d* = 1 pM for AD-2 peptide
Mechanism of Action	Blockade of gB-mediated viral internalization
Epitope Conservation	All published AD-2 sequences: 99% identical Activity shown against 15/15 clinical isolates
Cell Types Protected at Uniformly High Potency	Fibroblasts (HFF), Endothelial (HUVEC), Epithelial (ARPE), Placental Fibroblasts, Cytotrophoblasts, Trophoblast Progenitor Cells (TBPC), Dendritic Cells
Manufacturing	Stable CHO expression at 1.8 g/L at 100 L scale
28 day Toxicology (Sprague Dawley rat)	No Observed Adverse Effect Level: 150 mg/kg/dose
Immunogenicity	Expected to be low (native human mAb)
Half-life	46 hours in rats, projected to be 21 days in humans

## References

[1] Fulop T., Larbi A., Pawelec G. (2013). Human T cell aging and the impact of persistent viral infections. Front. Immunol..

[2] Pawelec G., McElhaney J.E., Aiello A.E., Derhovanessian E. (2012). The impact of CMV infection on survival in older humans. Curr. Opin. Immunol..

[3] Kotton C.N., Kumar D., Caliendo A.M., Asberg A., Chou S., Danziger-Isakov L., Humar A. (2013). Updated international consensus guidelines on the management of cytomegalovirus in solid-organ transplantation. Transplantation.

[4] De la Camara R. (2016). CMV in hematopoietic stem cell transplantation. Mediterr. J. Hematol. Infect. Dis..

[5] McCormick A.L., Mocarski E.S. (2015). The immunological underpinnings of vaccinations to prevent cytomegalovirus disease. Cell. Mol. Immunol..

[6] Chen K., Cheng M.P., Hammond S.P., Einsele H., Marty F.M. (2018). Antiviral prophylaxis for cytomegalovirus infection in allogeneic hematopoietic cell transplantation. Blood Adv..

[7] Emery V.C., Lazzarotto T. (2017). Cytomegalovirus in pregnancy and the neonate. F1000Research.

[8] Stagno S., Pass R.F., Cloud G., Britt W.J., Henderson R.E., Walton P.D., Veren D.A., Page F., Alford C.A. (1986). Primary cytomegalovirus infection in pregnancy. Incidence, transmission to fetus, and clinical outcome. JAMA.

[9] Turner K.M., Lee H.C., Boppana S.B., Carlo W.A., Randolph D.A. (2014). Incidence and impact of cmv infection in very low birth weight infants. Pediatrics.

[10] Zavattoni M., Lombardi G., Rognoni V., Furione M., Klersy C., Stronati M., Baldanti F. (2014). Maternal, fetal, and neonatal parameters for prognosis and counseling of HCMV congenital infection. J. Med. Virol..

[11] Yamamoto A.Y., Mussi-Pinhata M.M., Isaac Mde L., Amaral F.R., Carvalheiro C.G., Aragon D.C., Manfredi A.K., Boppana S.B., Britt W.J. (2011). Congenital cytomegalovirus infection as a cause of sensorineural hearing loss in a highly immune population. Pediatr. Infect. Dis. J..

[12] Barker D.J. (1999). Fetal origins of cardiovascular disease. Ann. Med..

[13] Iwasenko J.M., Howard J., Arbuckle S., Graf N., Hall B., Craig M.E., Rawlinson W.D. (2011). Human cytomegalovirus infection is detected frequently in stillbirths and is associated with fetal thrombotic vasculopathy. J. Infect. Dis..

[14] Syridou G., Spanakis N., Konstantinidou A., Piperaki E.T., Kafetzis D., Patsouris E., Antsaklis A., Tsakris A. (2008). Detection of cytomegalovirus, parvovirus b19 and herpes simplex viruses in cases of intrauterine fetal death: Association with pathological findings. J. Med. Virol..

[15] Kalil A.C., Freifeld A.G., Lyden E.R., Stoner J.A. (2009). Valganciclovir for cytomegalovirus prevention in solid organ transplant patients: An evidence-based reassessment of safety and efficacy. PLoS ONE.

[16] Pereira L., Tabata T., Petitt M., Fang-Hoover J. (2017). Congenital cytomegalovirus infection undermines early development and functions of the human placenta. Placenta.

[17] Tabata T., Petitt M., Fang-Hoover J., Rivera J., Nozawa N., Shiboski S., Inoue N., Pereira L. (2012). Cytomegalovirus impairs cytotrophoblast-induced lymphangiogenesis and vascular remodeling in an in vivo human placentation model. Am. J. Pathol..

[18] Rahav G. (2007). Congenital cytomegalovirus infection—A question of screening. Isr. Med. Assoc. J..

[19] Sijmons S., Van Ranst M., Maes P. (2014). Genomic and functional characteristics of human cytomegalovirus revealed by next-generation sequencing. Viruses.

[20] Stern-Ginossar N., Weisburd B., Michalski A., Le V.T., Hein M.Y., Huang S.X., Ma M., Shen B., Qian S.B., Hengel H. (2012). Decoding human cytomegalovirus. Science (New York).

[21] Sinzger C., Digel M., Jahn G. (2008). Cytomegalovirus cell tropism. Curr. Top. Microbiol. Immunol..

[22] Zhou M., Lanchy J.M., Ryckman B.J. (2015). Human cytomegalovirus gH/gL/gO promotes the fusion step of entry into all cell types, whereas gh/gl/ul128-131 broadens virus tropism through a distinct mechanism. J. Virol..

[23] Ryckman B.J., Rainish B.L., Chase M.C., Borton J.A., Nelson J.A., Jarvis M.A., Johnson D.C. (2008). Characterization of the human cytomegalovirus gH/gL/UL128-131 complex that mediates entry into epithelial and endothelial cells. J. Virol..

[24] Wang D., Shenk T. (2005). Human cytomegalovirus virion protein complex required for epithelial and endothelial cell tropism. Proc. Natl. Acad. Sci. USA.

[25] Dargan D.J., Douglas E., Cunningham C., Jamieson F., Stanton R.J., Baluchova K., McSharry B.P., Tomasec P., Emery V.C., Percivalle E. (2010). Sequential mutations associated with adaptation of human cytomegalovirus to growth in cell culture. J. Gen. Virol..

[26] Li G., Kamil J.P. (2016). Viral regulation of cell tropism in human cytomegalovirus. J. Virol..

[27] Zhang L., Zhou M., Stanton R., Kamil J., Ryckman B.J. (2018). Expression levels of glycoprotein o (go) vary between strains of human cytomegalovirus, influencing the assembly of gH/gL complexes and virion infectivity. J. Virol..

[28] Renzette N., Bhattacharjee B., Jensen J.D., Gibson L., Kowalik T.F. (2011). Extensive genome-wide variability of human cytomegalovirus in congenitally infected infants. PLoS Pathog..

[29] Kauvar L.M., Pereira L., Permar S., Kowalik T., Hamprecht K., Adler S.P., McVoy M.A. Trl345: A native human mab against cmv (gb: Ad-2 site I). Proceedings of the Cytomegalovirus Infection: Advancing Strategies for Prevention and Treatment, National Institute of Allergy and Infectious Diseases.

[30] Fisher C.E., Knudsen J.L., Lease E.D., Jerome K.R., Rakita R.M., Boeckh M., Limaye A.P. (2017). Risk factors and outcomes of ganciclovir-resistant cytomegalovirus infection in solid organ transplant recipients. Clin. Infect. Dis. Off. Publ. Infect. Dis. Soc. Am..

[31] Boeckh M., Nichols W.G., Chemaly R.F., Papanicolaou G.A., Wingard J.R., Xie H., Syrjala K.L., Flowers M.E., Stevens-Ayers T., Jerome K.R. (2015). Valganciclovir for the prevention of complications of late cytomegalovirus infection after allogeneic hematopoietic cell transplantation: A randomized trial. Ann. Intern. Med..

[32] Chan S.T., Logan A.C. (2017). The clinical impact of cytomegalovirus infection following allogeneic hematopoietic cell transplantation: Why the quest for meaningful prophylaxis still matters. Blood Rev..

[33] Foolad F., Aitken S.L., Chemaly R.F. (2018). Letermovir for the prevention of cytomegalovirus infection in adult cytomegalovirus-seropositive hematopoietic stem cell transplant recipients. Expert Rev. Clin. Pharmacol..

[34] Lischka P., Hewlett G., Wunberg T., Baumeister J., Paulsen D., Goldner T., Ruebsamen-Schaeff H., Zimmermann H. (2010). In vitro and in vivo activities of the novel anticytomegalovirus compound aic246. Antimicrob. Agents Chemother..

[35] Melendez D.P., Razonable R.R. (2015). Letermovir and inhibitors of the terminase complex: A promising new class of investigational antiviral drugs against human cytomegalovirus. Infect. Drug Resist..

[36] Marty F.M., Ljungman P., Chemaly R.F., Maertens J., Dadwal S.S., Duarte R.F., Haider S., Ullmann A.J., Katayama Y., Brown J. (2017). Letermovir prophylaxis for cytomegalovirus in hematopoietic-cell transplantation. N. Engl. J. Med..

[37] Goldner T., Hempel C., Ruebsamen-Schaeff H., Zimmermann H., Lischka P. (2014). Geno- and phenotypic characterization of human cytomegalovirus mutants selected in vitro after letermovir (aic246) exposure. Antimicrob. Agents Chemother..

[38] Chou S. (2015). Rapid in vitro evolution of human cytomegalovirus ul56 mutations that confer letermovir resistance. Antimicrob. Agents Chemother..

[39] Knoll B.M., Seiter K., Phillips A., Soave R. (2018). Breakthrough cytomegalovirus pneumonia in hematopoietic stem cell transplant recipient on letermovir prophylaxis. Bone Marrow Transplant..

[40] Lischka P., Michel D., Zimmermann H. (2016). Characterization of cytomegalovirus breakthrough events in a phase 2 prophylaxis trial of letermovir (aic246, mk 8228). J. Infect. Dis..

[41] Cherrier L., Nasar A., Goodlet K.J., Nailor M.D., Tokman S., Chou S. (2018). Emergence of letermovir resistance in a lung transplant recipient with ganciclovir-resistant cytomegalovirus infection. Am. J. Transplant. Off. J. Am. Soc. Transplant. Am. Soc. Transpl. Surg..

[42] Marty F.M., Ljungman P., Papanicolaou G.A., Winston D.J., Chemaly R.F., Strasfeld L., Young J.A., Rodriguez T., Maertens J., Schmitt M. (2011). Maribavir prophylaxis for prevention of cytomegalovirus disease in recipients of allogeneic stem-cell transplants: A phase 3, double-blind, placebo-controlled, randomised trial. Lancet Infect. Dis..

[43] Shire Efficacy and Safety Study of Maribavir Treatment Compared to Investigator-Assigned Treatment in Transplant Recipients with Cytomegalovirus (CMV) Infections That Are Refractory or Resistant to Treatment with Ganciclovir, Valganciclovir, Foscarnet, or Cidofovir. https://clinicaltrials.gov/ct2/show/NCT02931539.

[44] Shire Study for the Treatment of Cytomegalovirus (CMV) Infection in Hematopoietic Stem Cell Transplant Recipients. https://ClinicalTrials.gov/show/NCT02927067.

[45] Jabs D.A., Gilpin A.M., Min Y.I., Erice A., Kempen J.H., Quinn T.C. (2002). HIV and cytomegalovirus viral load and clinical outcomes in aids and cytomegalovirus retinitis patients: Monoclonal antibody cytomegalovirus retinitis trial. AIDS (London).

[46] Wu Y., Prager A., Boos S., Resch M., Brizic I., Mach M., Wildner S., Scrivano L., Adler B. (2017). Human cytomegalovirus glycoprotein complex gh/gl/go uses pdgfr-alpha as a key for entry. PLoS Pathog..

[47] Lin C.T., Hsueh P.R., Wu S.J., Yao M., Ko B.S., Li C.C., Hsu C.A., Tang J.L., Tien H.F. (2018). Repurposing nilotinib for cytomegalovirus infection prophylaxis after allogeneic hematopoietic stem cell transplantation: A single-arm, phase II trial. Biol. Blood Marrow Transplant. J. Am. Soc. Blood Marrow Transplant..

[48] Boppana S.B., Britt W.J. (2014). Recent approaches and strategies in the generation of antihuman cytomegalovirus vaccines. Methods Mol. Biol. (Clifton).

[49] McVoy M.A. (2013). Cytomegalovirus vaccines. Clin. Infect. Dis. Off. Publ. Infect. Dis. Soc. Am..

[50] Pass R.F., Zhang C., Evans A., Simpson T., Andrews W., Huang M.L., Corey L., Hill J., Davis E., Flanigan C. (2009). Vaccine prevention of maternal cytomegalovirus infection. N. Engl. J. Med..

[51] Lantto J., Fletcher J.M., Ohlin M. (2003). Binding characteristics determine the neutralizing potential of antibody fragments specific for antigenic domain 2 on glycoprotein b of human cytomegalovirus. Virology.

[52] Ohlin M. (2014). A new look at a poorly immunogenic neutralization epitope on cytomegalovirus glycoprotein B. Is there cause for antigen redesign?. Mol. Immunol..

[53] Griffiths P.D., Stanton A., McCarrell E., Smith C., Osman M., Harber M., Davenport A., Jones G., Wheeler D.C., O’Beirne J. (2011). Cytomegalovirus glycoprotein-b vaccine with mf59 adjuvant in transplant recipients: A phase 2 randomised placebo-controlled trial. Lancet (London).

[54] Sester M., Sester U., Gartner B., Kubuschok B., Girndt M., Meyerhans A., Kohler H. (2002). Sustained high frequencies of specific cd4 T cells restricted to a single persistent virus. J. Virol..

[55] Mori T., Kanda Y., Takenaka K., Okamoto S., Kato J., Kanda J., Yoshimoto G., Gondo H., Doi S., Inaba M. (2017). Safety of asp0113, a cytomegalovirus DNA vaccine, in recipients undergoing allogeneic hematopoietic cell transplantation: An open-label phase 2 trial. Int. J. Hematol..

[56] Samuel E.R., Beloki L., Newton K., Mackinnon S., Lowdell M.W. (2014). Isolation of highly suppressive cd25+foxp3+ t regulatory cells from g-csf-mobilized donors with retention of cytotoxic anti-viral ctls: Application for multi-functional immunotherapy post stem cell transplantation. PLoS ONE.

[57] Clark A.L., Gall S.A. (1997). Clinical uses of intravenous immunoglobulin in pregnancy. Am. J. Obstet. Gynecol..

[58] La Torre R., Nigro G., Mazzocco M., Best A.M., Adler S.P. (2006). Placental enlargement in women with primary maternal cytomegalovirus infection is associated with fetal and neonatal disease. Clin. Infect. Dis. Off. Publ. Infect. Dis. Soc. Am..

[59] Moise K.J., Wolfe H. (2008). Treatment of second trimester fetal cytomegalovirus infection with maternal hyperimmune globulin. Prenat. Diagn..

[60] Moxley K., Knudtson E.J. (2008). Resolution of hydrops secondary to cytomegalovirus after maternal and fetal treatment with human cytomegalovirus hyperimmune globulin. Obstet. Gynecol..

[61] Nigro G., Adler S.P., La Torre R., Best A.M. (2005). Passive immunization during pregnancy for congenital cytomegalovirus infection. N. Engl. J. Med..

[62] Nigro G., La Torre R., Anceschi M.M., Mazzocco M., Cosmi E.V. (1999). Hyperimmunoglobulin therapy for a twin fetus with cytomegalovirus infection and growth restriction. Am. J. Obstet. Gynecol..

[63] Revello M.G., Lazzarotto T., Guerra B., Spinillo A., Ferrazzi E., Kustermann A., Guaschino S., Vergani P., Todros T., Frusca T. (2014). A randomized trial of hyperimmune globulin to prevent congenital cytomegalovirus. N. Engl. J. Med..

[64] Adler S.P. (2012). Editorial commentary: Primary maternal cytomegalovirus infection during pregnancy: Do we have a treatment option?. Clin. Infect. Dis. Off. Publ. Infect. Dis. Soc. Am..

[65] Hamprecht K., Kagan K.O., Goelz R. (2014). Hyperimmune globulin to prevent congenital CMV infection. N. Engl. J. Med..

[66] Kagan K.O., Enders M., Schampera M.S., Baeumel E., Hoopmann M., Geipel A., Berg C., Goelz R., De Catte L., Wallwiener D. (2018). Prevention of maternal-fetal transmission of cmv by hyperimmunoglobulin (hig) administered after a primary maternal cmv infectionin early gestation. Ultrasound Obstet. Gynecol. Off. J. Int. Soc. Ultrasound Obstet. Gynecol..

[67] Hsu J.L., Safdar N. (2011). Polyclonal immunoglobulins and hyperimmune globulins in prevention and management of infectious diseases. Infect. Dis. Clin. N. Am..

[68] Bonaros N., Mayer B., Schachner T., Laufer G., Kocher A. (2008). Cmv-hyperimmune globulin for preventing cytomegalovirus infection and disease in solid organ transplant recipients: A meta-analysis. Clin. Transplant..

[69] Goldstein G., Rutenberg T.F., Mendelovich S.L., Hutt D., Oikawa M.T., Toren A., Bielorai B. (2017). The role of immunoglobulin prophylaxis for prevention of cytomegalovirus infection in pediatric hematopoietic stem cell transplantation recipients. Pediatr. Blood Cancer.

[70] Freed D.C., Tang Q., Tang A., Li F., He X., Huang Z., Meng W., Xia L., Finnefrock A.C., Durr E. (2013). Pentameric complex of viral glycoprotein h is the primary target for potent neutralization by a human cytomegalovirus vaccine. Proc. Natl. Acad. Sci. USA.

[71] Kabanova A., Marcandalli J., Zhou T., Bianchi S., Baxa U., Tsybovsky Y., Lilleri D., Silacci-Fregni C., Foglierini M., Fernandez-Rodriguez B.M. (2016). Platelet-derived growth factor-alpha receptor is the cellular receptor for human cytomegalovirus ghglgo trimer. Nat. Microbiol..

[72] Fouts A.E., Chan P., Stephan J.P., Vandlen R., Feierbach B. (2012). Antibodies against the gh/gl/ul128/ul130/ul131 complex comprise the majority of the anti-cytomegalovirus (anti-cmv) neutralizing antibody response in cmv hyperimmune globulin. J. Virol..

[73] Revello M.G., Fornara C., Arossa A., Zelini P., Lilleri D. (2014). Role of human cytomegalovirus (hcmv)-specific antibody in hcmv-infected pregnant women. Early Hum. Dev..

[74] Dupont L., Reeves M.B. (2016). Cytomegalovirus latency and reactivation: Recent insights into an age old problem. Rev. Med. Virol..

[75] Macagno A., Bernasconi N.L., Vanzetta F., Dander E., Sarasini A., Revello M.G., Gerna G., Sallusto F., Lanzavecchia A. (2010). Isolation of human monoclonal antibodies that potently neutralize human cytomegalovirus infection by targeting different epitopes on the gh/gl/ul128–131a complex. J. Virol..

[76] Scrivano L., Sinzger C., Nitschko H., Koszinowski U.H., Adler B. (2011). HCMV spread and cell tropism are determined by distinct virus populations. PLoS Pathog..

[77] Manley K., Anderson J., Yang F., Szustakowski J., Oakeley E.J., Compton T., Feire A.L. (2011). Human cytomegalovirus escapes a naturally occurring neutralizing antibody by incorporating it into assembling virions. Cell Host Microbe.

[78] Ishida J.H., Patel A., Mehta A.K., Gatault P., McBride J.M., Burgess T., Derby M.A., Snydman D.R., Emu B., Feierbach B. (2017). Phase 2 randomized, double-blind, placebo-controlled trial of rg7667, a combination monoclonal antibody, for prevention of cytomegalovirus infection in high-risk kidney transplant recipients. Antimicrob. Agents Chemother..

[79] Deng R., Wang Y., Maia M., Burgess T., McBride J.M., Liao X.C., Tavel J.A., Hanley W.D. (2018). Pharmacokinetics and exposure-response analysis of rg7667, a combination of two anticytomegalovirus monoclonal antibodies, in a phase 2a randomized trial to prevent cytomegalovirus infection in high-risk kidney transplant recipients. Antimicrob. Agents Chemother..

[80] Ohlin M., Sundqvist V.A., Mach M., Wahren B., Borrebaeck C.A. (1993). Fine specificity of the human immune response to the major neutralization epitopes expressed on cytomegalovirus gp58/116 (gb), as determined with human monoclonal antibodies. J. Virol..

[81] Patel H.D., Nikitin P., Gesner T., Lin J.J., Barkan D.T., Ciferri C., Carfi A., Akbarnejad Yazdi T., Skewes-Cox P., Wiedmann B. (2016). In vitro characterization of human cytomegalovirus-targeting therapeutic monoclonal antibodies ljp538 and ljp539. Antimicrob. Agents Chemother..

[82] Dole K., Segal F.P., Feire A., Magnusson B., Rondon J.C., Vemula J., Yu J., Pang Y., Pertel P. (2016). A first-in-human study to assess the safety and pharmacokinetics of monoclonal antibodies against human cytomegalovirus in healthy volunteers. Antimicrob. Agents Chemother..

[83] McCutcheon K.M., Gray J., Chen N.Y., Liu K., Park M., Ellsworth S., Tripp R.A., Tompkins S.M., Johnson S.K., Samet S. (2014). Multiplexed screening of natural humoral immunity identifies antibodies at fine specificity for complex and dynamic viral targets. mAbs.

[84] Potzsch S., Spindler N., Wiegers A.K., Fisch T., Rucker P., Sticht H., Grieb N., Baroti T., Weisel F., Stamminger T. (2011). B cell repertoire analysis identifies new antigenic domains on glycoprotein b of human cytomegalovirus which are target of neutralizing antibodies. PLoS Pathog..

[85] Bialas K.M., Westreich D., Cisneros de la Rosa E., Nelson C.S., Kauvar L.M., Fu T.M., Permar S.R. (2016). Maternal antibody responses and nonprimary congenital cytomegalovirus infection of hiv-1-exposed infants. J. Infect. Dis..

[86] Boppana S.B., Britt W.J. (1995). Antiviral antibody responses and intrauterine transmission after primary maternal cytomegalovirus infection. J. Infect. Dis..

[87] Maidji E., McDonagh S., Genbacev O., Tabata T., Pereira L. (2006). Maternal antibodies enhance or prevent cytomegalovirus infection in the placenta by neonatal fc receptor-mediated transcytosis. Am. J. Pathol..

[88] Utz U., Britt W., Vugler L., Mach M. (1989). Identification of a neutralizing epitope on glycoprotein gp58 of human cytomegalovirus. J. Virol..

[89] Meyer H., Sundqvist V.A., Pereira L., Mach M. (1992). Glycoprotein gp116 of human cytomegalovirus contains epitopes for strain-common and strain-specific antibodies. J. Gen. Virol..

[90] Collarini E.J., Lee F.E., Foord O., Park M., Sperinde G., Wu H., Harriman W.D., Carroll S.F., Ellsworth S.L., Anderson L.J. (2009). Potent high-affinity antibodies for treatment and prophylaxis of respiratory syncytial virus derived from b cells of infected patients. J. Immunol..

[91] Harriman W.D., Collarini E.J., Sperinde G.V., Strandh M., Fatholahi M.M., Dutta A., Lee Y., Mettler S.E., Keyt B.A., Ellsworth S.L. (2009). Antibody discovery via multiplexed single cell characterization. J. Immunol. Methods.

[92] Kauvar L.M., Liu K., Park M., DeChene N., Stephenson R., Tenorio E., Ellsworth S.L., Tabata T., Petitt M., Tsuge M. (2015). A high-affinity native human antibody neutralizes human cytomegalovirus infection of diverse cell types. Antimicrob. Agents Chemother..

[93] Finnefrock A.C., Freed D.C., Tang A., Li F., He X., Wu C., Nahas D., Wang D., Fu T.M. (2016). Preclinical evaluations of peptide-conjugate vaccines targeting the antigenic domain-2 of glycoprotein b of human cytomegalovirus. Hum. Vaccines Immunother..

[94] Janeway C.A., Travers P., Walport M., Shlomchik M.J. (2001). Immunobiology: The Immune System in Health and Disease.

[95] Thomson C.A., Bryson S., McLean G.R., Creagh A.L., Pai E.F., Schrader J.W. (2008). Germline v-genes sculpt the binding site of a family of antibodies neutralizing human cytomegalovirus. EMBO J..

[96] Jackson K.J., Liu Y., Roskin K.M., Glanville J., Hoh R.A., Seo K., Marshall E.L., Gurley T.C., Moody M.A., Haynes B.F. (2014). Human responses to influenza vaccination show seroconversion signatures and convergent antibody rearrangements. Cell Host Microbe.

[97] Parameswaran P., Liu Y., Roskin K.M., Jackson K.K., Dixit V.P., Lee J.Y., Artiles K.L., Zompi S., Vargas M.J., Simen B.B. (2013). Convergent antibody signatures in human dengue. Cell Host Microbe.

[98] Ishibashi K., Tokumoto T., Shirakawa H., Hashimoto K., Ikuta K., Kushida N., Yanagida T., Shishido K., Aikawa K., Toma H. (2011). Lack of antibodies against the antigen domain 2 epitope of cytomegalovirus (cmv) glycoprotein b is associated with cmv disease after renal transplantation in recipients having the same glycoprotein h serotypes as their donors. Transpl. Infect. Dis..

[99] Hackett D.J., Zhang C., Stefanescu C., Pass R.F. (2010). Enzyme-linked immunosorbent assay for measurement of cytomegalovirus glycoprotein b antibody in serum. Clin. Vaccine Immunol..

[100] Meyer-Konig U., Haberland M., von Laer D., Haller O., Hufert F.T. (1998). Intragenic variability of human cytomegalovirus glycoprotein b in clinical strains. J. Infect. Dis..

[101] Fu T.-M., Tang A., Wang D., An Z., Zhang N., Ha S. (2017). CMV Neutralizing Antigen Binding Proteins.

[102] Cui X., Adler S.P., Schleiss M.R., Arav-Boger R., Demmler Harrison G.J., McVoy M.A. (2017). Cytomegalovirus virions shed in urine have a reversible block to epithelial cell entry and are highly resistant to antibody neutralization. Clin. Vaccine Immunol..

[103] Mariame B., Kappler-Gratias S., Kappler M., Balor S., Gallardo F., Bystricky K. (2018). Real-time visualization and quantification of human cytomegalovirus replication in living cells using the anchor DNA labeling technology. J. Virol..

[104] Cui X., Freed D.C., Wang D., Qiu P., Li F., Fu T.M., Kauvar L.M., McVoy M.A. (2017). Impact of antibodies and strain polymorphisms on cytomegalovirus entry and spread in fibroblasts and epithelial cells. J. Virol..

[105] Fisher S., Genbacev O., Maidji E., Pereira L. (2000). Human cytomegalovirus infection of placental cytotrophoblasts in vitro and in utero: Implications for transmission and pathogenesis. J. Virol..

